# Electronic reminders to facilitate longitudinal care: a mixed-methods study in general practices

**DOI:** 10.1186/s12911-016-0387-z

**Published:** 2016-11-24

**Authors:** Christine Kersting, Birgitta Weltermann

**Affiliations:** Institute for General Medicine, University Hospital Essen, University of Duisburg-Essen, Hufelandstr. 55, 45147 Essen, Germany

**Keywords:** Mixed methods, Longitudinal care, Primary health care, Reminder system, Health information technology, Electronic health record

## Abstract

**Background:**

Longitudinal, patient-centered care represents a challenge for general practitioners (GPs), and in this context, reminder systems can offer targeted support. This study aimed to identify details of such reminders: (1) contents of care addressed, (2) their mode of display in the electronic health record (EHR), (3) their visual appearance, (4) personnel responsibilities for editing and applying reminders, and (5) use of reminders for patient recall.

**Methods:**

This mixed-methods study comprised (1) a cross-sectional survey among 185 GP practices from a German university network, and (2) structured observations of reminder utilization in six practices based on a clinical vignette describing a multimorbid senior with 26 care needs. Descriptive statistics were performed for survey data. The practice observations were analyzed by portraying different types of reminders.

**Results:**

Seventy-three of 185 practices completed the survey (39.5%): 98.6% reported using reminders in the EHR. Frequent care contents addressed were allergies/adverse drug events (95.8%), preventive measures (93.1%), participation in disease management programs (87.5%), chronic diseases (75.0%), and upcoming vaccinations (68.1%). Practice observations showed a variety of mainly self-configured reminders. In a patients’ EHR, information was displayed (1) compiled in a separate field, (2) scattered throughout the EHR, and/or (3) in a pop-up window. The visual appearance of electronic reminders varied: (1) colored fields with short text, (2) EHR entries and/or billing codes in pre-defined colors, (3) abbreviations within the treatment documentation, (4) symbols within the treatment documentation, (5) symbols linked to free text fields, and (6) traffic light schemes. Five practices self-designed reminders ‘as needed’; one practice applied an EHR-embedded, pre-defined reminder system. Practices used reminders for a mean of 13.3 of the 26 aspects of care detailed in the clinical vignette (range: 9–21; standard deviation (SD): 4.3). Practices needed 20–35 min (mean: 27.5; SD: 6.1) to retrieve the information requested.

**Conclusions:**

Most GP practices use self-designed, visual reminders for some aspects of care, yet data-based, sophisticated solutions are needed to improve longitudinal care.

**Trial registration:**

German Clinical Trials Register, unique identifying number: DRKS00008777 (date of registration: 06/19/2015).

**Electronic supplementary material:**

The online version of this article (doi:10.1186/s12911-016-0387-z) contains supplementary material, which is available to authorized users.

## Background

A 2014 consensus statement of four professional organizations of family physicians and pediatricians addressed gaps in the functionality of current electronic health record (EHR) systems. The organizations called for a change in focus from electronic documentation to evidence-based, comprehensive, and patient-centered whole-person care. Such an approach should allow for longitudinal tracking of information, which is a prerequisite for adequately addressing the complexity and variety of patients [1]. This shift is urgently needed in view of the aging society and the complexity of care for multimorbid seniors. Serial cross-sectional surveys conducted by the Commonwealth Fund showed that current EHR solutions are not designed to adequately support the management of complex patients [2–5]. A 2012 comparison of eleven countries revealed that EHR implementation varied largely: whereas only 41% of general practitioners (GPs) in Switzerland and 57% in Canada routinely used EHR, the implementation rate was about 70% in France and in the United States, about 80% in Germany, and above 90% in Australia, New Zealand, Sweden, Norway, the United Kingdom, and the Netherlands [5]. An unusual discrepancy was discovered in German and Norwegian GP practices: while the EHR implementation rate was high, the multifunctional capacity of the available health information technology was low [2]. Only 11% of German GPs reported routinely using computerized, guideline-based reminders, while 18% use computer-based reminders to recall patients [4, 5]. By definition, reminder systems systematically provide prompts or hints to recall information or advice the user is aware of, but can be easily forgotten [6], for example upcoming vaccinations and examinations. These systems may therefore contribute to solutions addressing patients’ complexity.

Current efforts to support primary care by health information technology typically focus on individual chronic diseases [7–10]. A Cochrane Review with 28 studies on on-screen, point of care reminders documented small, but significant improvements of process- and patient-related outcomes [6]. Compared to usual care, overall median improvements in process adherence, e.g., test ordering or recommended vaccinations associated with reminders, accounted for 5.7%, while those of patient-centered clinical outcomes were lower [6]. Studies on reminder systems to facilitate the management of patients with more than one chronic disease also showed improvements in quality of care [11–13], but these studies are still underrepresented.

Given the limited functionalities of current EHR solutions and unsystematic observations that some GPs develop their own reminders, this mixed-methods study aimed to describe details of reminder systems applied in German GP practices. In the future, this information will be used to develop better software for improving the longitudinal care of complex patients in GP practices.

## Methods/design

### Study design

Aiming at a comprehensive understanding of the use of reminders in GP practices, we performed a mixed-methods study with a concurrent design which equally involved (1) quantitative and (2) qualitative methods [14, 15]:

1. Quantitative study: To quantify reminder utilization in GP practice, we conducted a *cross-sectional survey* among the 185 GP practices from the practice network of the Institute for General Medicine, University of Duisburg-Essen, Germany.
2. Qualitative study: To describe the actual design and use of reminders in GP practices, a scientific researcher of the institute performed structured *process observations* of EHR reminders in six network practices that volunteered for the study.

The study is part of a larger-scale study focusing on the development of a software solution, which aims at supporting longitudinal care in GP practices. Details of the objectives and methods are published elsewhere [16].

### Practice recruitment

For the quantitative study (*cross-sectional survey)*, a questionnaire was mailed to all 185 GP practices of the university practice network. For the qualitative study *(process observations)* all network practices that participated in the networks fall meeting in September 2015 (*n* = 124 GPs) were informed about the process observations. Twelve physicians from ten practices volunteered; these practices were then contacted and invited to participate.

### Data collection

#### Quantitative data

The *cross-sectional survey* was performed using a three-page written questionnaire, which asked for (1) the contents of care addressed by reminders, and (2) the patient groups recalled by reminders (Additional file 1). Data on practice characteristics were available for all practices of the network as every GP practice joining the university practice network is required to complete a basic questionnaire on practice characteristics.

#### Qualitative data

The *structured process observations* were conducted by one of the researchers (C.K.). To structure the process observations we had constructed a clinical vignette describing a complex multimorbid senior with 26 aspects of care in various categories (chronic diseases, upcoming preventive measures, geriatric testing, required follow-up examinations, lifestyle characteristics, functional impairments, participation in special care contracts, and persons involved into patients’ care processes) (Table 1). First, this clinical vignette was explained to the participating practice personnel, then they were asked to demonstrate if and how they document each respective aspect of care within each EHR. In detail, we requested (1) the contents of care addressed by reminders, (2) their mode of display in the EHR, (3) their visual appearance, (4) personnel responsibilities for editing and applying reminders, and (5) for which patient groups recall systems are used. The process demonstrations were performed by a practice assistant with or without a GP being present. This approach is reasonable for the German health care system, because practice assistants assume tasks in practice organization and patient management. These assistants have completed a certified 3-year vocational training. The observations were documented using a standardized, semi-structured documentation sheet containing a checklist of all 26 aspects of care addressed as well as free text fields allowing for detailed descriptions of the reminders’ visual appearance and mode of display. The time required to retrieve the information requested was recorded in all practices.

**Table 1:** Clinical vignette of a multimorbid senior for the structured process observations

Sex	Male
Age	76 years
Health insurance	Statutory health insurance^a^
Chronic diseases	- Type 2 diabetes mellitus - Hypertension - Atrial fibrillation - Hashimoto
Participation in special care contracts	- GP-centered care - DMP type 2 diabetes mellitus
Prevention & functional testing	- Check-up (bi-yearly) - Skin cancer screening (bi-yearly) - Prostate cancer screening (yearly) - Geriatric assessment (yearly) - Diphtheria booster (every 10 years) - Tetanus booster (every 10 years) - Influenza immunization (yearly)
Follow-up examinations	- Repeat colonoscopy in 2019 is recommended (colonoscopy 2016: tubular adenoma) - Control of INR - DMP diabetes examinations (twice a year) - Diabetes feet check (yearly) - Referral for diabetic eye check (yearly) - Thyroid control
Lifestyle characteristics	Smoker
Characteristics/impairments	- Receives oral anticoagulants - Poor hearing and poor eyesight - Sometimes nagging - The patient’s advance health care directive is deposited in the practice
Persons involved in care processes	- Mobile nursing service that helps the patient to put on the compression stockings to prevent leg ulcer in venous insufficiency - Next of kin has to be involved in care decisions

^a^Nearly every person living in Germany has a health insurance, either private or statutory. About 90 % are members of the statutory health insurance (sickness funds), which is financed predominantly by contributions paid by employers and employees

#### Quality controls and data management

Consent forms of all study participants were checked for completeness. Handwritten documentations of the process observations were transcribed. The quantitative survey data were entered manually in an access-restricted database. All data are stored access-restricted at the institute.

### Data analysis

Data of the cross-sectional survey and of the process observations were analyzed separately from each other. Quantitative survey data and checklist items from the process observations were analyzed using descriptive statistics in IBM SPSS Statistics for Windows, Version 22. Frequencies and mean values are reported for valid cases. To ensure a reliable analysis of the process observations, two researchers (B.W., C.K.) described similarities and differences of the various reminders with regard to their visual appearance and their mode of display. Based on these descriptions, both researchers agreed on portrays for different types of reminders. To assure external validity, the portrays were compared to the literature on reminders.

Following these analyses, quantitative and qualitative data were integrated. Data were merged by way of constructing a joint display (table) which allowed for interpretation and discussion to gain a deeper understanding of reminder utilization in German GP practices [14, 15, 17].

## Results

### Cross-sectional survey

#### Practice characteristics

Seventy-three of the 185 GP practices completed the survey (39.5%). The majority of these practices were group practices (*n* = 42; 63.6%) (Table 2). On average, 2.5 physicians (standard deviation (SD): 1.6) and 5.7 practice assistants (SD: 4.9) were employed in these practices. Participating practices did not differ from non-participants with regard to practice setting, number of GPs and practice assistants per practice, and patient volume per quarter. Detailed information is provided in Table 2.

**Table 2:** Practice characteristics

	Cross-sectional survey (*N* = 73)	Process observations (*N* = 6)
Practice setting, *n* (%)		
* Solo practice*	*23 (34.8)*	*1 (16.7)*
* Group practice*	*42 (63.6)*	*5 (83.3)*
* Other*	*1 (1.4)*	*0 (0)*
Number of physicians, mean ± SD (range)	2.5 ± 1.6 (1–10)	1.8 ± 0.8 (1–3)
Number of practice assistants, mean ± SD (range)	5.7 ± 4.9 (1–35)	4.5 ± 1.4 (2–6)
Number of patients in practice per quarter, *n* (%)		
* 501 to 1000*	*9 (14.1)*	*0 (0)*
* 1001 to 1500*	*14 (21.9)*	*3 (50.0)*
* 1501 to 2000*	*15 (23.4)*	*1 (16.7)*
* 2001 to 2500*	*6 (9.4)*	*1 (16.7)*
* 2501 to 3000*	*6 (9.4)*	*0 (0)*
* >3000*	*14 (21.9)*	*1 (16.7)*

Percentages and mean values are reported for valid cases

*Abbreviation*: *SD* standard deviation

#### Utilization of reminders

Of the 73 practices, 72 (98.6%) reported using reminders in the paper-based health records (HR) and/or EHR to keep an overview of each patient’s care. The most frequent aspects of care addressed by reminders were: allergies and/or adverse drug events (69 of 72; 95.8%), preventive measures, e.g. cancer screening, and check-up (in Germany, persons aged ≥35 years are entitled to a bi-yearly health examination focusing on cardiovascular risks) (67 of 72; 93.1%), patients’ participation in a disease management program (DMP) (63 of 72; 87.5%), practice-individual selected chronic conditions (54 of 72; 75.0%), and vaccinations (49 of 72; 68.1%).

Reminder systems for patient recalls were used by 54 practices (74.0%), especially for patients participating in a DMP (48 of 54; 88.9%). Other reasons for recall were upcoming preventive measures (33 of 54; 61.1%), vaccinations (22 of 54; 40.7%), and follow-up examinations (20 of 54; 37.0%). More details are provided in Table 3.

**Table 3:** Reminders used to support patient-centered health care management in German GP practices

Cross-sectional survey (*N* = 72)		Process observations (*N* = 6)		
Content of care	*N* (%)	Content of care	*N* (%)	Description of the reminders’ visual appearance *(mode of display)*
Chronic diseases	54 (75.0)	Chronic diseases	3 (50.0)	- Combination of a colored box and a short text, i.e. ‘diabetes’ *(permanently displayed in a separate field)* - Diagnosis-related entries and/or billing codes in pre-defined colors *(scattered/ requires interaction)*
Chronic medication	48 (66.7)	Oral anticoagulants	5 (83.3)	- Combination of a colored box and a short text, i.e. ‘marcumar’ *(permanently displayed in a separate field)* - ‘red hand’ symbol with a free text field detailing relevant patient information *(permanently displayed in a separate field)* - Billing codes in pre-defined colors *(scattered/ requires interaction)* - Provision of relevant aspects of care including information on anticoagulant medication when opening a patient’s EHR *(pop-up window)* - Handwritten information on the cover of the paper-based HR *(paper-based reminder)*
Lifestyle characteristics	40 (55.6)	Smoker	2 (33.3)	- Combination of colored box and a short text, i.e. ‘smoker’ *(permanently displayed in a separate field)* - ‘i’ symbol with a free text field detailing relevant patient information *(permanently displayed in a separate field)*
Patient specifics	34 (46.6)	Impairment	4 (66.7)	- Combination of colored box and a short text, i.e. ‘poor hearing’ *(permanently displayed in a separate field)* - ‘i’ symbol with a free text field detailing relevant patient information *(permanently displayed in a separate field)* - ‘light bulb’ symbol linked to a free text field detailing relevant patient information when clicking on it *(scattered/ requires interaction)* - Provision of relevant aspects of care including information on ability to see/hear when opening a patient’s EHR *(pop-up window)*
		“Soft skills”, e.g., aggressive	2 (33.3)	- Combination of colored box and a short text, i.e. ‘aggressive’ *(permanently displayed in a separate field)* - ‘red hand’ symbol with a free text field detailing relevant patient information *(permanently displayed in a separate field)*
Allergies/ adverse events	69 (95.8)	*Not assessed*	–	
Upcoming follow-up examinations	41 (56.9)	Re-colonoscopy after 3 years	5 (83.3)	- Combination of colored box and a short text, i.e. ‘colonoscopy 2018’ *(permanently displayed in a separate field)* - Tagging of a postdated entry within the electronic treatment documentation using a stop sign *(permanently displayed in a separate field)* - Tagging of entries within the electronic treatment documentation with a specific abbreviation, i.e. ‘WV’, or an exclamation mark allowing searches *(scattered/ requires interaction)* - ‘light bulb’ symbol linked to a free text field detailing relevant patient information when clicking on it *(scattered/ requires interaction)* - Provision of relevant aspects of care including information on upcoming follow-up examinations when opening a patient’s EHR *(pop-up window)*
		Sonography of the thyroid	3 (50.0)	- Combination of colored box and a short text, i.e. ‘sono’ *(permanently displayed in a separate field)* - Tagging of entries within the electronic treatment documentation with a specific abbreviation, i.e. ‘sono’, allowing searches *(scattered/ requires interaction)* - ‘light bulb’ symbol linked to a free text field detailing relevant patient information when clicking on it *(scattered/ requires interaction)* - Provision of relevant aspects of care including information on regular follow-up examinations when opening a patient’s EHR *(pop-up window)*
Upcoming preventive measures	67 (93.1)	Screening tests/check-up	4 (66.7)	- Combination of colored box and a short text, i.e. ‘check-up 2016’ *(permanently displayed in a separate field)* - Tagging of entries within the electronic treatment documentation with a specific abbreviation, i.e. ‘gu’, allowing searches *(scattered/ requires interaction)* - ‘to do’ symbol linked to a free text field detailing relevant patient information when clicking on it *(scattered/ requires interaction)* - List of all preventive measures that can be billed at the moment *(pop-up window)* - Handwritten information on the cover of the paper-based HR *(paper-based reminder)*
		Geriatric assessment	2 (33.3)	- Combination of colored box and a short text, i.e. ‘geriatric assessment 2016’ *(permanently displayed in a separate field)* - Tagging of entries within the electronic treatment documentation with a specific abbreviation, i.e. ‘egs’, allowing searches *(scattered/ requires interaction)* - Provision of a list of all preventive measures that can be billed at the moment *(pop-up window)*
Upcoming vaccinations	49 (68.1)	Upcoming vaccinations	3 (50.0)	- Tagging of entries within the electronic treatment documentation with a specific abbreviation, i.e. ‘imp’, allowing searches *(scattered/ requires interaction)* - Traffic light scheme indicating the need for vaccination *(scattered/ requires interaction)*
Self-pay services	8 (11.1)	*Not assessed*	–	
DMP participation	63 (87.5)	DMP participation	4 (66.7)	- Combination of colored box and a short text, i.e. ‘DMP diabetes’ *(permanently displayed in a separate field)* - Billing codes in pre-defined colors *(scattered/ requires interaction)* - Handwritten information on the cover of the paper-based HR *(paper-based reminder)*
*Not assessed*	–	Participation in GP-centered care	4 (66.7)	- Combination of colored box and a short text, i.e. ‘GP-centered care’ *(permanently displayed in a separate field)* - Field naming the patients’ health insurance company in pre-defined colors *(permanently displayed in a separate field)* - Pop-up window indicating if a patient is participating or is eligible to participate in GP-centered care *(pop-up window)*
*Not assessed*	–	Next of kin’s involvement to care decisions	5 (83.3)	- Combination of colored box and a short text, i.e. ‘caretaker’ *(permanently displayed in a separate field)* - ‘i’ symbol with a free text field detailing relevant patient information *(permanently displayed in a separate field)* - ‘light bulb’ symbol linked to a free text field detailing relevant patient information when clicking on it *(scattered/ requires interaction)* - Provision of relevant aspects of care including information on caretaker when opening a patient’s EHR *(pop-up window)* - Handwritten contact information on the cover of the paper-based HR *(paper-based reminder)*
*Not assessed*	–	Mobile nursing service	2 (33.3)	- Provision of relevant aspects of care including information on mobile nursing when opening a patient’s EHR *(pop-up window)* - Handwritten contact information on the cover of the paper-based HR *(paper-based reminder)*
*Not assessed*	–	Storage of patients’ advance health care directive in the practice	2 (33.3)	- Combination of a colored box and a short text, i.e. ‘advance health care directive’ *(permanently displayed in a separate field)*

### Structured process observations

#### Practice characteristics

Six practices participated in the process observations. All participating practices used reminders. Three of the practices (50.0%) were group practices with an average of 1.8 physicians (SD: 0.8) and 4.5 practice assistants (SD: 1.4) (Table 2). The 3-month patient volume varied between the practices: three practices cared for 1001 to 1500 patients, one practice for 1501 to 2000 patients, one for 2001 to 2500 patients, and the largest practice provided services to more than 3000 patients. See Table 2 for details.

In four of the practices the structured process observation was conducted with a practice assistant and in two practices with a GP and a practice assistant. The majority of practices worked purely electronically; only one practice used a combination of HR and EHR. Of the roughly 100 EHR solutions available on the German market, the six participating GP practices used four different solutions. These EHR solutions vary by the interface design and the options for reminders. On average, 27.5 min (range: 20 to 35, SD: 6.1) were needed to retrieve the 26 aspects of care requested in the clinical vignette. In five of the six practices (83.3%) a vast number of user-software interactions were necessary to retrieve the requested overview; only one practice used an EHR-embedded, pre-defined reminder system that provided the information requested in pre-defined fields on one screen.

#### Description of reminders

On average, the six practices used reminders for 13.3 aspects of care (SD: 4.3, range: 9–21) of the 26 aspects outlined in the vignette (Table 3). The contents of care most commonly addressed by reminders were treatment with oral anticoagulants (*n* = 5; 83.3%), necessity to involve the patients’ next of kin in care decisions (*n* = 5; 83.3%), and repeat colonoscopy for intestinal polyps (tubular adenoma) after 3 years (*n* = 5; 83.3%). The next frequent aspects of care indicated by a reminder were upcoming preventive measures (prostate cancer screening, check-up, and skin cancer screening, each *n* = 4; 66.7%), participation in a special care contract (DMP, GP-centered care, each *n* = 4; 66.7%), and patients’ impairments (*n* = 4; 66.7%).

**Fig. 1:**
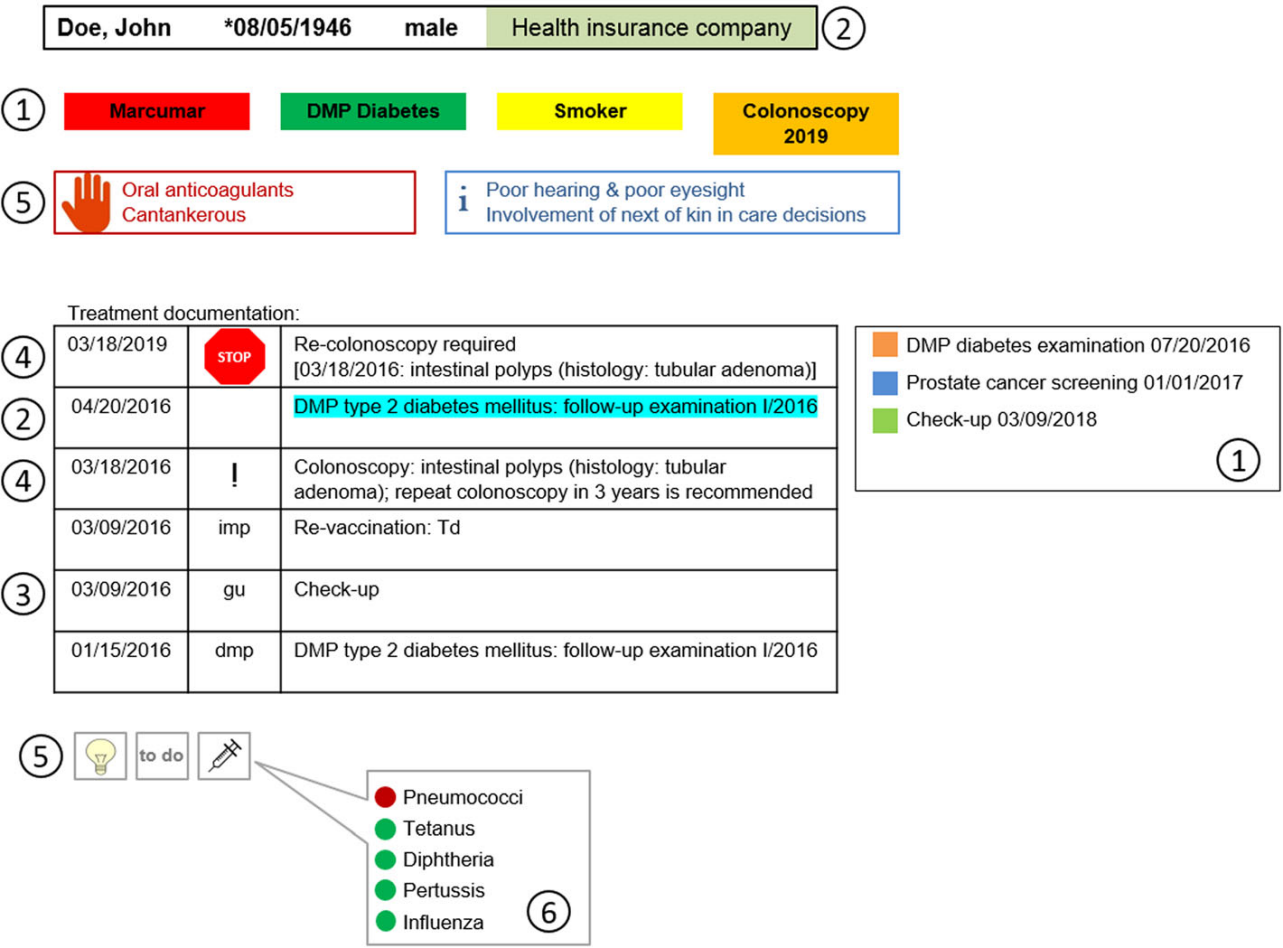
Stylized illustration of the electronic reminders (Category 1 to 6 of the visual appearances identified; see ‘Descriptions of reminders’)

The information was displayed (1) compiled in a separate field, which was permanently displayed in each patient’s EHR, (2) scattered throughout the EHR requiring interaction to retrieve the information, and/or (3) in pop-up windows providing information when opening a patient’s EHR. The following visual appearances of electronic reminders were identified (Fig. 1):

1. Combination of a colored box and a short text in the EHR, i.e. providing information on important aspects of patients’ care and/or upcoming follow-up examinations.
2. Diagnosis-related EHR entries, billing codes, and/or other relevant care aspects (e.g., contract specifics) in pre-defined colors.
3. Tagging of entries within the physicians’ treatment documentation using searchable specific abbreviations, i.e. ‘dmp’.
4. Tagging of entries within the physicians’ treatment documentation using a specific symbol, i.e. a stop sign or an exclamation mark.
5. Symbols linked to a free text field allowing for unstructured patient-related entries, i.e. a light bulb, an ‘i’, or a red hand.
6. Colors as used by German traffic lights indicating the need for vaccination (red: lack of vaccinations; yellow: vaccinations incomplete; green: vaccinations completed).

The practice that worked with a combination of HR and EHR used a paper-based reminder system: reminders were provided as handwritten short texts on the cover of the HR, detailing upcoming follow-up examinations and/or preventive measures or important aspects of care.

In two practices (33.3%), reminders were edited and applied under the responsibility of one designated person (GP or practice assistant). In the four other practices, all personnel were allowed to apply reminders.

Four of the six practices (66.7%) used recalls for defined patient groups and contents of care (Table 4). The most common reasons to recall patients were upcoming health examinations and/or check-ups (3 of 4), DMP-related examinations (2 of 4), and vaccinations (1 of 4).

**Table 4:** Reminders used to recall patients in German GP practices

	Cross-sectional survey (*N* = 73)	Process observations (*N* = 6)
Practices using reminders to recall their patients, *n* (%)	54 (74.0)	4 (66.7)
Reasons for reminder utilization, *n* (%):		
* DMP-related contents of care*	*48 (88.9)*	*2 (50.0)*
* Preventive measures/check-ups*	*33 (61.1)*	*3 (75.0)*
* Vaccinations*	*22 (40.7)*	*1 (25.0)*
* Follow-up examinations*	*20 (37.0)*	*0 (0)*
* Patients with chronic conditions which are poorly controlled*	*14 (25.9)*	*0 (0)*
* Patients on chronic medication*	*10 (18.5)*	*0 (0)*
* Self-pay services*	*1 (1.9)*	*0 (0)*

## Discussion

In our study, nearly all GP practices used an EHR-based reminder system for patient care. Currently, most reminders are used to recall contents of care, which are also relevant for billing, e.g. preventive measures, check-ups, or DMP-related contents of care. Interestingly, the set-up of reminder systems varied largely between practices: (1) from few reminders to comprehensive reminder systems, (2) from occasional to systematic display of reminders, and (3) from simple to sophisticated designs. We saw that the number and kind of reminders and reminder systems vary even between practices that use the same EHR software solution. Interestingly, all practices bar one self-designed their reminders as needed for a specific context of care, yet neither necessarily comprehensive, nor based on epidemiology. Similar reminder systems as portrayed in our study are described in other studies: reminders are delivered by systems used in routine care, are accessible by pop-up screens or icons within the software solution routinely used, address clinical and process-related care aspects, and target the personnel responsible for health care provision [6].

In contrast to the Commonwealth Fund studies [2–5], we observed higher rates of reminder utilization in German GP practices (reminder system for GPs: 11% versus 99–100%; utilization of reminders for patient recall: 18% versus 67–74% [4, 5]). This result is easily explained by the difference in the methodological approaches used: while the cross-sectional Commonwealth Fund studies were based on standardized telephone interviews and questionnaires, we performed a mixed-methods study including structured process observations, which allow for more detailed and more reliable descriptions of health information technology utilization. Unlike the Commonwealth Fund studies, our study was not limited to standard functionalities provided by EHR solutions [2–5], but also assessed practice-individual, self-designed approaches including HR-based approaches. However, the fact that practices self-design reminder systems is certainly a consequence of the low multifunctional capacity of German EHR systems described by the Commonwealth Fund studies [2].

Our approach and results are difficult to compare with other studies because there are no data on the actual design of reminder systems. Available intervention trials used different approaches as they evaluated the effectiveness of newly implemented, often imposed reminder systems in GP practices rather than focusing on the development of existing systems [6–13]. Also, most of the reminder systems studied support the management of a single chronic disease rather than the management of patient-centered, longitudinal chronic care across diseases [6–10].

### Strengths and limitations

The results of our study are limited due to a potential selection bias: it cannot be excluded that responders were more interested in health information technology and/or were more technophile than non-responders. Also, GP practices that use reminder systems on a daily basis might have been more willing to participate than those who do not, although the frequency of reminder utilization was not assessed. The key strength of this study is the mixed-methods approach: unlike purely quantitative approaches, the qualitative process observations are more reliable to assess the design, functionalities, and utilization of reminder systems in detail. Nevertheless, the time to retrieve the information outlined in the clinical vignette might be inaccurate because of the study situation.

## Conclusion and perspectives

In agreement with the 2014 consensus statement of Krist et al. [1] and the 2009 Commonwealth Fund study [2] we saw (1) that current EHR systems focus on documentation and billing rather than on supporting comprehensive, longitudinal, and patient-centered health care management, and (2) that the multifunctional capacity of EHR systems used in German GP practices is limited. Interestingly, practices generate self-defined reminders as needed to overcome this limitation. We are currently conducting semi-structured focus group interviews with GPs and practice assistants to assess their needs and requirements for a software solution, which supports evidence-based and longitudinal health care management for complex patients. In the future, it will be important to explore the effects of such software solution on the quality and integration of care as well as on patient-centered decision making.

## Additional file

Additional file 1: Questionnaire which was used for the cross-sectional survey among GP practices: it assessed the contents of care addressed by reminders and the patient groups recalled. (PDF 286 kb)

## Abbreviations

DMP: Disease management program
EHR: Electronic health record
GP: General practitioner
HR: Health record
INR: International normalized ratio
SD: Standard deviation
