# Supplementary material for: Electronic reminders to facilitate longitudinal care: a mixed-methods study in general practices
Source: BMC Med Inform Decis Mak. 2016 Nov 24;16:148. doi: 10.1186/s12911-016-0387-z (PMC5122020; doi:10.1186/s12911-016-0387-z)
Supplement: Additional file 1: — Questionnaire which was used for the cross-sectional survey among GP practices: it assessed the contents of care addressed by reminders and the patient groups recalled. (PDF 286 kb) [file 12911_2016_387_MOESM1_ESM.pdf]

Institute for General Medicine
University Hospital Essen
University of Duisburg-Essen
Hufelandstraße 55
45147 Essen

ADDITIONAL FILE 1:

Survey

'Management of care in general practices'

1. **Does your practice use any markers/reminders in the electronic health records and/or in the paper-based health records to get reminded on upcoming examinations and/or important contents of care? Please provide detailed information.** ***(Multiple answers allowed)***

   ☐$_0$ no

   ☐$_1$ yes, for:

   ☐$_1$ Allergies/adverse events, namely:____________________________________
   ____

   ☐$_1$ Preventive measures/check-ups, namely:______________________________
   ____

   ☐$_1$ Vaccinations, namely:____
   ____

   ☐$_1$ DMP participation, namely: ____
   ____

   ☐$_1$ Follow-up examinations, namely:____________________________________
   ____

   ☐$_1$ Chronic diseases, namely:____
   ____

   ☐$_1$ Chronic medication, namely:____
   ____

   ☐$_1$ Lifestyle characteristics (e.g., nicotine, alcohol, drugs), namely:____________
   ____

   ☐$_1$ Other patient specifics, namely:______________________________________
   ____

   ☐$_1$ Self-pay services, namely:____
   ____

   ☐$_1$ Other aspects:____

2. **Does your practice keep checklists/routine lists for any contents of care? Please provide detailed information.** ***(Multiple answers allowed)***

☐$_0$ no

☐$_1$ yes:

☐$_1$ For billing purposes, namely:____

____

☐$_1$ For risk management, namely:____

____

☐$_1$ For patient management, namely:_____________________________________

____

☐$_1$ For other purposes:____

3. **Does your practice use recall for defined contents of care and/or patient groups? Please provide detailed information.** ***(Multiple answers allowed)***

☐$_0$ no

☐$_1$ yes, for:

☐$_1$ Preventive measures/check-ups, namely:______________________________

____

☐$_1$ Vaccinations, namely:____

____

☐$_1$ DMP-related contents of care, namely:________________________________

____

☐$_1$ Follow-up examinations, namely:____________________________________

____

☐$_1$ Patients with chronic conditions which are poorly controlled, namely:_______

____

☐$_1$ Patients on chronic medication, namely:______________________________

____

☐$_1$ Self-pay services, namely:____

____

☐$_1$ Other care contents/patient groups:__________________________________

Thank you very much for participating!
